# Mapping energy citizenship in the south of Europe

**DOI:** 10.3389/fpsyg.2023.1112457

**Published:** 2023-08-09

**Authors:** Adina Claudia Dumitru, Luisa Losada-Puente, Manuel Peralbo, Juan Carlos Brenlla, Nuria Rebollo-Quintela, Manuel García-Fernández

**Affiliations:** ^1^Department of Psychology, Faculty of Educational Studies, University of A Coruña, A Coruña, Spain; ^2^Department of Specific Didactics and Methods of Research and Diagnosis in Education, Faculty of Educational Studies, University of A Coruña, A Coruña, Spain

**Keywords:** energy citizenship, social innovation, sustainable transition, energy governance, citizen empowerment, energy democracy

## Abstract

The adoption of new global approaches in the field of energy democratization requires inquiring into how people act to shape the energy system. This is where the concept of energy citizenship (ENCI) appears as a constellation of actors that enable and/or support citizens to became active participants in the debates and energy systems both in private and public sphere, or as a collective citizen that contributes to change (Pel et al., 2021). The aim of this paper is to explore the concept of ENCI in Southern Europe. Using a mixed approach, an extensive mapping of 43 ENCI initiatives in Spain (*n* = 29) and Portugal (*n* = 14) was conducted through desktop research, and a stakeholder consultation workshop (*n* = 7) was carried out through a focus group. Results revealed the major presence of collective ENCI types, with the *citizen-based/hybrid* one standing out (e.g., energy cooperatives). Most of them were motivated by the interest to contribute to energy transition or to produce and/or use renewable energy, and aimed at promoting energy saving, energy justice and reducing the carbon footprint. The general tendency is towards active participation (in Spain) and transformative forms (in Portugal). The possibilities for citizen control in the initiatives analysed is still limited. These results were confirmed by stakeholders who, additionally, pointed out the major political, social, economic, and geographical factors related to ENCI forms. In short, various types of ENCI could be validated in the Spanish and Portuguese context, showing a commitment to sustainability, democracy, and energy justice. Other non-evidenced forms may be raised as a challenge to further in-depth research on latent forms of ENCI in Southern Europe.

## 1. Introduction

The complex challenges of climate change, environmental degradation, and biodiversity loss have long signalled the need for fast-paced sustainability transitions. Such transitions imply profound changes in our current systems of production and consumption, including food, mobility, energy, and the built environment (Vita et al., 2020). The last Intergovernmental Panel on Climate Change (IPCC) report signalled that ambitious targets are needed to maintain global temperatures below 1.5 degrees’ increase, compared to 1990 levels, and pointed to the need for systemic social and cultural transformations, alongside technological innovation to reach them (Intergovernmental Panel on Climate Change, 2022).

Transforming energy systems and behaviours is an important goal of the European Green Deal, through the Renewables Directive (RED II) and the Internal Electricity Market Directive (IEMD) which introduce both the *prosumer* and a framework to facilitate the development of energy communities. The shift towards renewable energy sources (RES) and the need for deep transformations in energy production and consumption patterns have also led to a reconceptualization of the role of different actors in the energy system, from one that is centralized, dominated by experts and characterized by top-down decision-making, to one that is de-centralized, democratic and horizontal, and in which citizens engage actively and assume a variety of roles and responsibilities.

The concept of energy *citizenship* (hereafter ENCI) has been proposed as a construct that emphasizes more active participation in the shaping of the energy system, towards more sustainable and democratic forms (Devine-Wright, 2007). It has evolved both as a scientific concept and as an imaginary of active and engaged citizens who democratically and collaboratively shape the energy system (Pel et al., 2021). It alludes to the idea of normative commitments and responsibilities in sustainability transformations and responds to a policy desire to build broader acceptability for the fast-paced and often quite radical changes needed stay within planetary boundaries (Rockström et al., 2009). It also points to a certain normative ideal of an enlightened, ecologically-minded citizenship (Pel et al., 2021).

Taking a critical perspective, scholars have argued that traditional forms of energy system organization tend to rely on a social representation of the citizen as passive, and lacking the interest, knowledge, and responsibility to participate in shaping the energy system. An alternative representation emerged that framed energy as a social necessity and the citizen as an active participant in its transformation (Devine-Wright, 2007). Although the neoliberal and more radical forms of ENCI share a core vision of the active citizen, they differ on the prominence they assign to the private versus public spheres of action and to the consideration they give to power relations and the limitations they impose on individual agency (Lennon et al., 2020). While the active-passive distinction refers to behaviour or action, in its ideal version, the concept of ENCI involves particular assumptions regarding individual capacities and profiles. Levels of knowledge, awareness, motivations to act, and skills are assumed. Also, the ideal energy citizen engages in political action in a continuum between the private and the public sphere, and between the system-confirming and system-opposing extremes.

Such a representation entails, in the private sphere, a vision of the energy citizen as someone who expresses herself politically through conscious consumerism. She makes purchase decisions that consider the energy footprint of products, and installs house technologies that contribute to energy efficiency, through monitoring and actions to reduce overall consumption. Within the public sphere, she pays attention to energy debates and expresses positions actively through voting, or the lending of support to various initiatives and movements (e.g., signing petitions, maybe joining a manifestation). Such a perspective has been criticized as reflecting a neoliberal perspective of the citizen as consumer and a constraining public sphere of participation, where questions of exclusion and inequality are avoided (Lennon et al., 2020). A less constraining perspective on citizenship focuses on the active energy citizen as deeply engaged in participation processes to shape the energy transition. A certain type of compliant participation, whereby citizens endorse the basic tenets of the centralised energy system and/or its pathways towards change or transformation (e.g., by focusing the debate on where to install solar panels or windmills) is treated as desirable, with the overall objectives centred on endorsement or acceptability of the top-down policies. In this predominantly top-down approach, participation is equated to active engagement within an agenda set by public officials and endorsed by technical experts.

In contrast, advocates for a bottom-up approach to participation, and an enlarged sphere for citizen engagement with energy system transformation (Lennon et al., 2020) place their empirical and policy focus on the higher-commitment required in starting or joining of energy cooperatives, becoming a prosumer, or joining social movements to change energy systems, lifestyles, or patterns of production and consumption. This type of energy citizen endorses ideals of sustainability, believes in her capacity to act on goals that matter to her (Avelino et al., 2020) and becomes creative in generating the social relations and systems that can support a relocation of citizenship within wider contexts of social engagement in the energy system (Defila et al., 2018; Mullally et al., 2018), as notions such as social innovation have proposed. The active versus passive distinction of ENCI places the emphasis on the acting (knowledgeable and caring) individual, versus the non-acting (ignorant, disinterested) individual. From a business as usual, system-confirming perspective, acting includes classical political behaviour, with its private versus public sphere variants, with empirical manifestations such as conscious consumerism, voting, participating in public consultations and even joining an energy cooperative to have access to RES. From a system transformation or social innovation perspective, acting involves a deeper questioning of existing structures for engagement and participation, and actions or behaviours to enlarge the potential for action, through engagement in collectives and social movements.

This, in turn, has raised the questions of forms of energy citizenship encountered in practice and conditions of, and barriers to, active participation and empowerment of citizens. ENCI has come to include both individual and collective forms of manifestation (Defila et al., 2018; Mullally et al., 2018; Lennon et al., 2020). In the individual sphere, environmentally friendly energy behaviours, decisions to invest in energy efficient and smart technology, to shift to RES, become a prosumer or be an activist for the transformation of the energy systems have been included as manifestations of ENCI. In the collective sphere, manifestations such as energy communities, energy-related social innovation movements, as well as political militancy for the transformation of the energy system, have been considered. Furthermore, the multiplicity of types of engagement with the energy system raises the question of the extent to which they actually have the ambition or manage to contribute to energy system transformation in practice.

Research on social innovations in the energy domain has highlighted their transformative ambitions for empowerment, challenging of power relations and incorporating values of community, solidarity, and authenticity in the organization of the energy system (Wittmayer et al., 2022). Social innovation has been defined as changes in social relations that involves new ways of knowing, doing, organizing, and framing (Pel et al., 2021). Empirical research on social innovations has looked at the impacts they seek, in terms of changes in more or less institutionalized social relations, values, as well as practices and behaviours, which have been highlighted as more important than only focusing on changes in the organization and institutionalized decision-making structures of the energy system (Avelino et al., 2020). Moreover, environmental awareness, critical insight and the capacity for political mobilization have also been signalled as important outcomes of participatory energy movements and communities (Smith et al., 2016; Pel et al., 2022a).

Citizen participation in the energy system is not an all-or-nothing issue, so it is not possible to refer to what is and is not an energy citizen, nor to a single typology of ENCI (Pel et al., 2021) as it will depend on various factors that define in each context “new roles and responsibilities of citizens in an energy system in a constant transformation” (Van Wees et al., 2022, p. 1). Notable examples of ENCI in the framework of democratising the energy system through social innovations include energy-related grassroots initiatives (Hewitt et al., 2019), which stand out for their potential to promote individual and collective capacity to meeting certain social needs and achieve sustainability goals (Vita et al., 2020; Strasser et al., 2022); for example, by generating and distributing renewable energy whose benefits accrue to citizens themselves; by proposing alternative forms of individual or collective living and housing that are self-sufficient; by generating new forms of participation in the public sphere; and by mobilizing public opinion and voting in favour of energy transition policy measures.

Several authors refer to the concept of ENCI as a useful construct to differentiate the various forms of citizen participation in the energy transition (Campos and Marín-González, 2020) or the adoption of different roles in the energy system (e.g., as consumer, protester, supporter, or prosumer) (Ringholm, 2022; van Wees et al., 2022). ENCI might thus take different forms in practice, and empirical manifestations might differ from the ideal type forms described above (Devine-Wright, 2007).

**Figure 1:**
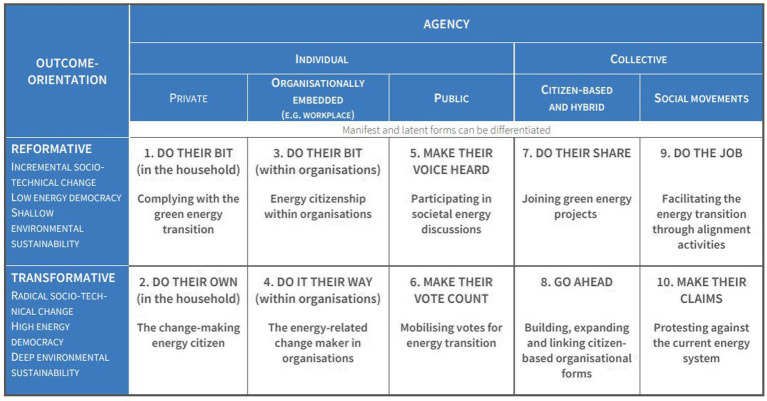
Overview of the ten types of ENCI. Extracted from Debourdeau et al. (2021, p. 35).

To make sense of this diversity, and to guide empirical research, a typology of ideal-type forms of ENCI was developed, using two key organizing dimensions: agency whether they take the form of individual commitments, values and behaviours, or to collective forms of participation in the energy system, such as energy cooperatives or communities aiming to promote energy system transformation; and their result-orientation, which can be either reformative or transformative, understood here in terms of the nature of the aims they pursue (Pel et al., 2022a). The typology includes ten ideal-type cases of energy citizenship (Figure 1). Through these ideal types it is possible to describe different forms of energy citizenship, ranging from manifest and visible forms to latent forms, which can only be detected empirically (Debourdeau et al., 2021; Pel et al., 2021, 2022a). All these concepts should be understood within a continuum, and not as opposing poles under which ENCI types are based.

Building on this typology, the present paper zooms in on the empirical manifestations in Southern Europe, in particular in Spain and Portugal. The latest European Parliament Barometer reports (European Commission, 2021) reveal that European citizens consider climate change as one of the most serious problems in the world, followed by poverty, hunger and lack of water. It is ranked as a priority in Northern and Central European countries (e.g., Denmark, Sweden, and the Netherlands), and is also higher than in previous reports in southern European countries such as Spain, Portugal, and Italy. Furthermore, recent data from the Barometer of Attitudes towards the Environment (European Commission, 2020) place Spain and Portugal in the middle of the EU-27 in considering environmental protection as a very important element and show higher concern than in previous years. In Spain, citizens tend to be in favour of the energy transition and the low-carbon economy, although they leave the ultimate responsibility to national governments (67%), companies and industry (60%) or the EU itself (58%), and to a lesser extent, they assume their own responsibility for action (42%), or that of environmental groups (32%). Similarly, in Portugal it is a priority to be addressed mainly by the EU (64%), government (57%) and industry (48%), and to a lesser extent by citizens (43%) and environmental groups (23%).

Despite such apparently promising numbers, some studies have concluded that countries such as Spain or Portugal are lagging behind in terms of energy transition, democratization, and citizen empowerment (Hewitt et al., 2019) as well as in the involvement of civil society in the energy transition, more broadly (Soeiro and Ferreira, 2020).

The present paper aims to identify and analyse the types of energy citizenship that can be encountered in the south of Europe on the basis of different approaches: on the one hand, exploring the characteristics (such as motivations and objectives for their development, agency and outcome orientation) of the ENCI types in the Spanish and Portuguese context; and on the other hand, putting the focus on a particular Spanish region, near to Portugal in order to analyse the social, political, economic and geographical factors that favour or hinder the development of the aforementioned ENCI types.

## 2. Methodology

We used a mixed methodological design to identify the forms of energy citizenship and their characteristics in Spain and Portugal and to understand the conditions that foster or hinder different forms in this region of Europe.

Previous research has identified different types of energy initiatives present in EU countries. These previous efforts, such as the database created in the H2020 Energise project (Jensen et al., 2017) or cases studied in other EU projects on energy transitions and social innovations were used as a starting point to create a comprehensive catalogue of approximately 600 ENCI initiatives in Europe.

For the selection of the initiatives, unified criteria were established by the consortium: only European initiatives were included, meaning those active either in the European Union, in the EEA or in ascension countries; and that were currently active or had concluded after 2015, when the EU Energy Strategy was published. Five criteria for the selection of cases were used: (a) type of geographical area covered by the initiative (rural, peri-urban and urban); (b) main focus of the initiative, differentiating between those whose direct focus is energy production and consumption, those who address mobility, and those who try to foster sustainable lifestyles from a more holistic perspective, with indirect effects on energy system transformation; (c) type of agency exhibited by the case, either individual or collective and the sphere in which it operates (private, organisational, public, citizenship-based or hybrid, and social movements); (d) outcome orientation, either reformative or transformative, and (e) attention paid to gender and issues of equity, more broadly.

The initiatives selected as meeting the above criteria were mapped through their websites and official documents. The H2020 Energy Prospects consortium designed a survey (Survey Monkey, Vadovics et al., 2022) related to the 5-level approach to identify and classify the different cases and to describe their characteristics. A set of research questions were formulated for each of the cases. These were included in the online survey tool and case researchers were asked to provide an answer based on extended analyses of case documents. The questions included: basic information about the case and the documents used to analyse it; basic characteristics of the case; its objectives and motivations, results, and progress towards objectives; the evolution of the case, including its start, changes over time and reasons for termination, if the case; the actors involved; the governance structure, functioning and funding; and specific questions regarding the theoretical ENCI typology to test (i.e., individual/collective agency, and reformative/transformative outcome orientation).

To both validate and deepen the analysis on the regional characteristics of the different ENCI cases encountered (Pel et al., 2022b) a stakeholder consultation workshop was held energy citizenship experts and stakeholders in Galicia (a Spanish region bordering the Portuguese territory) with which, historically, it has been twinned for reasons of language, proximity, and transnational cooperation.

The purpose of this workshop was to refine the ideal ENCI typologies with key informants, for which seven mainly non-academic ENCI experts and practitioners were selected to discuss key aspects of their experiences with the typology developed in Debourdeau et al. (2021). This workshop was carried out simultaneously by several partners in different regions (Berlin, Germany; Budapest, Hungary; and Galicia, Spain; and Wallonia, Belgium). A common script was used by the different partners; that is: *could you identify two regional examples of ENCI; could you identify one example of ENCI for each type of agency? could you identify two examples of reformative/transformative ENCI? what are the social, political, economic, and geographical factors that favour/hinder the emergence of ENCI types in the region?*

For the selection of informants, a series of steps were carried out: first, an extensive review of different governmental organisations, companies, and various intermediary institutions in Galicia and with a high level of knowledge on the subject (more or less explicitly) was carried out. A database was created with the most relevant information regarding 42 organisations and their representatives (names, contacts, location); secondly, the database was filtered to seek representativeness of the three sectors: Government, intermediaries of the Civil Society and Business; thirdly, contact was established by e-mail with 3–4 agents for each of the three sectors. Finally, seven experts from all over Galicia participated and signed their consent to record the meeting to facilitate its subsequent transcription and authorised the use of the data derived from the event. Each participant had a high-decision making level in his/her organisation, included one of the biggest energy providers, two officials from local and regional public administration and one from a regional delegation of a national centre, one member of a R&D centre, and two representatives of institutions in the construction sector, one of them coming from a non-profit association.

The workshop was held in one morning, from 11 am-14 pm. It was structured in such a way that, at the beginning, members of the research team gave an overview of the project and the ENCI typology (Figure 1) just to structure and focus the workshops. Through the common questions, discussion was opened, and a broader systematic exploration was possible. Conceptual categories were further explored in the context of the social, cultural, economic, and geographical conditions that give rise to ENCI forms. Six people moderated the three-hour workshop in a hybrid format. The role of the moderators was that of modest facilitators, alternating short introductions to the topics with individual and group work and collective discussions afterwards.

## 3. Results

Results that are presented below give an overview of the representative and visible ENCI types in the Spanish and Portuguese territories. Note that the theoretical model of ideal ENCI types tries to reflect all the possibilities of agency and outcomes, but the aim of this study is just to verify which of these ideal types fit with the reality studied. So, this mapping was carried out under demanding criteria (see *methodology*) and, as a result, it has revealed initiatives that are manifested in the territory. Latent initiatives may have remained hidden, as well as some others that do not have information available to the public (necessary for the mapping) or that are not known by people involved in the Galician region (stakeholders’ workshop).

### 3.1. Results of energy citizenship mapping

The Spanish and Portuguese mapping included 43 cases distributed throughout the peninsular and insular territory. Table 1 shows a comparison between both countries in terms of potential ENCI initiatives by geographical area covered and aim.

**Table 1:** Potential Spanish and Portuguese ENCI initiatives by geographical location and aim.

		Spain		Portugal		Total	
		*n*	%	*n*	%	*n*	%
No. ENCI cases		29	67.44	14	32.56	43	100
Geography	Rural	7	77.78	2	22.22	9	20.93
	Peri-urban area	4	100	0	0	4	9.30
	Urban area	4	57.14	3	42.85	7	16.28
	Several/all of above	12	60	8	40	20	46.51
	Non-relevant distinction (virtual case)	2	66.67	1	33.33	3	6.97
Main focus	Direct energy production/consumption	15	71.43	6	28.57	21	2.33
	Mobility	0	0	1	100	1	48.84
	Holistic/focus on broader change	14	66.67	7	33.33	21	48.84

Initiatives covering several geographical areas (rural, peri-urban, and urban) were the most prominent in both Spain (*n* = 12) and Portugal (*n* = 8). As for their aim, initiatives were considered if they fell into one of the following categories: (a) focusing specifically on energy consumption and production, (b) on mobility (car-free living, cycling related cases, travel less, no-flight initiatives), or (c) fostering a holistic perspective to sustainable lifestyle change with implications for energy production and consumption (carbon footprint reduction, communities, sufficiency-oriented cases). Initiatives directly related to production and consumption were the most prominent in Spain (*n* = 15), while holistic cases stood out in Portugal (*n* = 7), as well as one case related to mobility.

In terms of the types of agency characterizing the initiatives, a small percentage of them were individual cases (scientists who become activists, individuals who care about carbon footprint reduction and are an example of this, influencers, people who live self-sufficiently) and the majority were of the collective type (citizens as minority shareholders in a wind or solar farm project; ECs or cooperatives; non-profit organizations promoting debate on and acceptance of transmission power lines and grid development; climate protest movements). However, access to information regarding individual cases was challenging, as many were not sufficiently documented to carry out desk-based mapping. Thus, most of the initiatives identified in the mapping were collective types, with only two cases of individual agency in Spain (6.89%) and none for Portugal.

Table 2 presents the distribution of the potential ENCI according to the type of agency (individual/collective) and the outcome orientation of the initiatives (reformative/transformative), as well as within the former, the sphere in which it operates (private, organisational, public, citizenship-based or hybrid, and social movements). Citizen-based/hybrid ENCI type was the most prominent in both countries. This type of collective agency stood out both for initiatives with reformative and transformative outcomes. Also, some private initiatives (individual and reformative) were found, especially in the case of Spain. At the organisational level, no noteworthy initiatives were found in Portugal, and few were found in Spain. Social movements stood out slightly more in Portugal. There were no initiatives analysed whose main typology was considered public (individual). Only in the Spanish case was there one initiative that was considered, secondarily, as a possible individual - public reformatory type (e.g., Granada in Transition). As for the focus on issues related to disadvantaged groups (e.g., those in fuel poverty, minorities, etc.), five of the 43 initiatives were found to be of this concern, all of them from Spain. Other initiatives may be addressing this issue in a less focused way.

**Table 2:** Potential Spanish and Portuguese ENCI initiatives according to the type of agency and outcomes orientation.

		Type of agency												
		Individual							Collective					
Outcome orientation		Spain		Portugal		Total			Spain		Portugal		Total	
		*n*	%	*n*	%	*N*	%		*n*	%	*n*	%	*N*	%
Reformative	Private sphere	7	70	3	30	10	23.8	Citizen-based/hybrid	5	55.6	4	44.4	9	21.4
	Organizational sphere	3	100			3	7.1	Social movement	2	66.7	1	33.3	3	7.1
Transformative	Private sphere	2	66.7	1	33.33	3	7.1	Citizen-based/hybrid	7	63.6	4	36.4	11	26.2
	Organizational sphere	1	100			1	2.4	Social movement	1	50	1	50	2	4.8
	Totals					17	40.4						25	59.6

**Figure 2:**
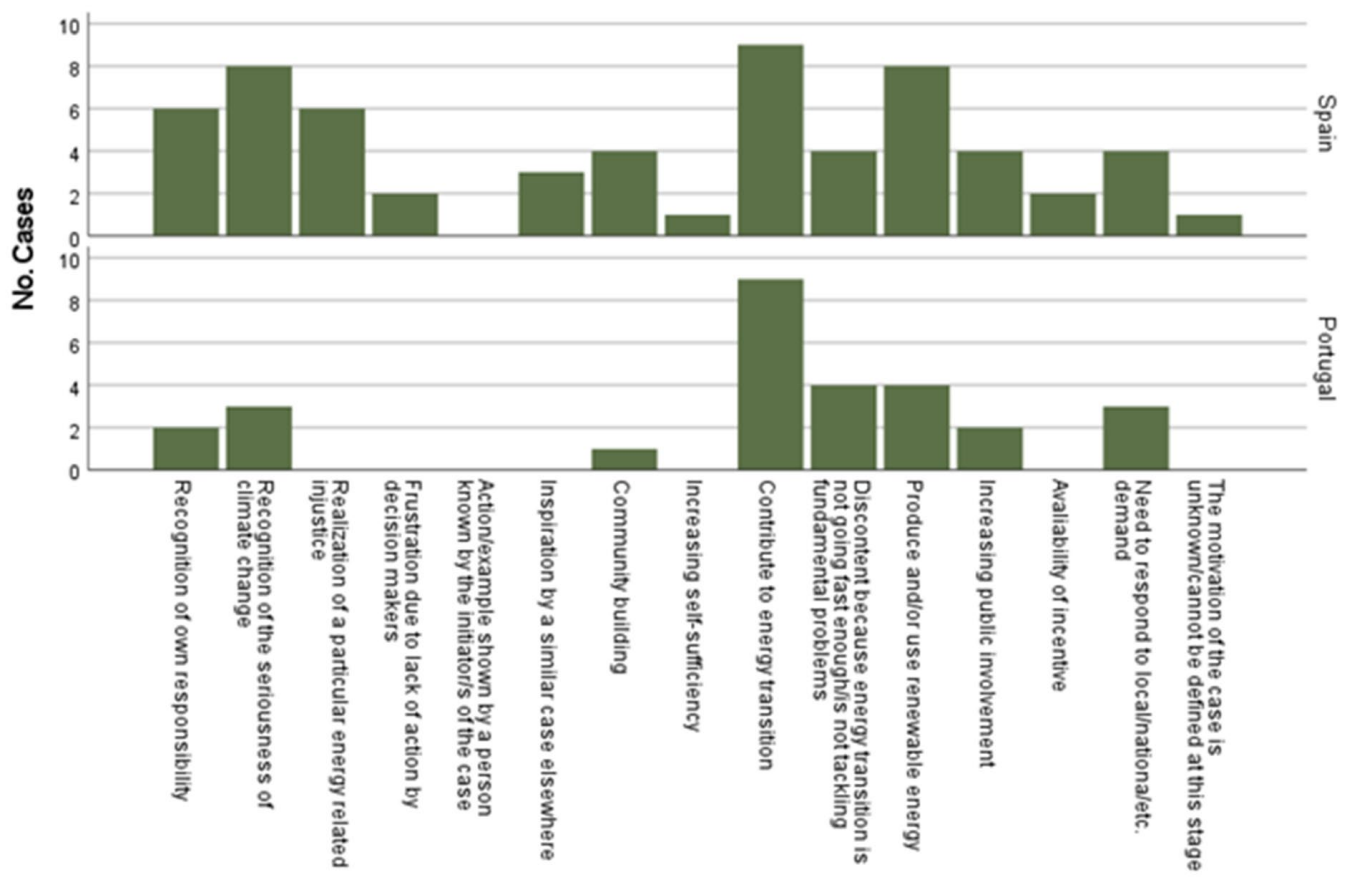
Main motivations for initiating the case in the Spanish and Portuguese ENCI initiatives. Own elaboration.

No cases of individual agency in the public sphere were obtained, so this table does not include ENCI Types 5 and 6 referred to in Figure 2.

Regarding the empirical manifestations of ENCI in Southern Europe^1^, generally the Spanish and Portuguese initiatives that stand out are those initiated by interested citizens (e.g., cooperatives, social protest movements); others are also promoted by public bodies after observing one or more needs in a group of people (e.g., electrification programmes in isolated areas, installation of renewable energy plants in specific locations) or in society (e.g., educational projects, dissemination, and information to citizens). The highest proportion of initiatives have been created in the last 15 years: *n* = 18 (41.9%) between 2011 and 2015 and *n* = 7 (16.3%) between 2016 and 2020 and in equal number between 2006 and 2010. In Spain, the greatest boost to ENCI initiatives occurred in 2011–2015 (*n* = 14, 48.3%) and in Portugal, in equal proportion between 2011–2015 and 2016–2020 (*n* = 4, 28.6%).

**Figure 3:**
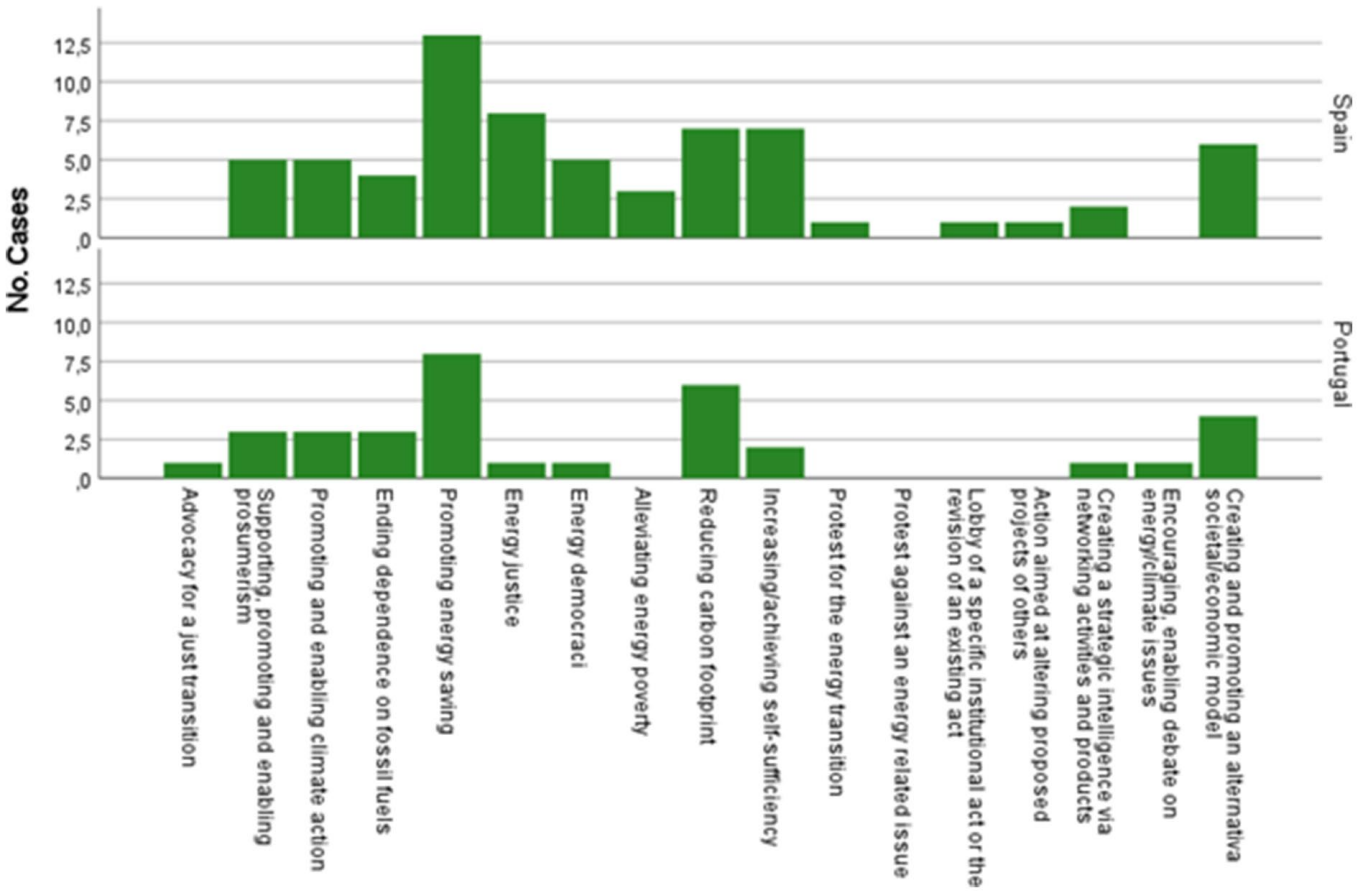
Main objectives/goals for initiating the case in the Spanish and Portuguese ENCI initiatives. Own elaboration.

Figures 2, 3 graphically represent the main motivations and objectives/ambitions in both contexts (see Supplementary material of information on each initiative).

As shown in Figure 2, in both countries, the main motivation for ENCI initiatives is the contribution to the energy transition (e.g., ZERO or PEGADAS in Portugal; EOLPOP or Madrid 100% Sostenible in Spain), followed also in both cases as one of the second motivations the production and/or use of renewable energy (e.g., Som Energía, GoiEner in Spain; and EnergizAir and DecoProteste in Portugal). In Spain, the second main motivation was also found to be the recognition of the seriousness of climate change (e.g., Energia Comunitaria); while in Portugal, discontent that the energy transition is not going fast enough (e.g., EuroToptenAct). No cases have been found that have, as their main motivation, examples of previous initiatives initiated by other people, and only in one Spanish case (O Couso Project) the definition of their motivations was not explicit from the beginning.

In terms of the objectives/ambitions that guided the creation of these initiatives, Figure 3 highlights the promotion of energy saving (e.g., BEHAVE and TRIBE in Spain - although these initiatives were also implemented in Portugal; and EnergizAir in Portugal), followed in the Spanish case by energy justice (e.g., Energy Audits of Friends of the Earth Spain), and in the Portuguese case, by the reduction of the carbon footprint (e.g., EcoCommunities).

**Figure 4:**
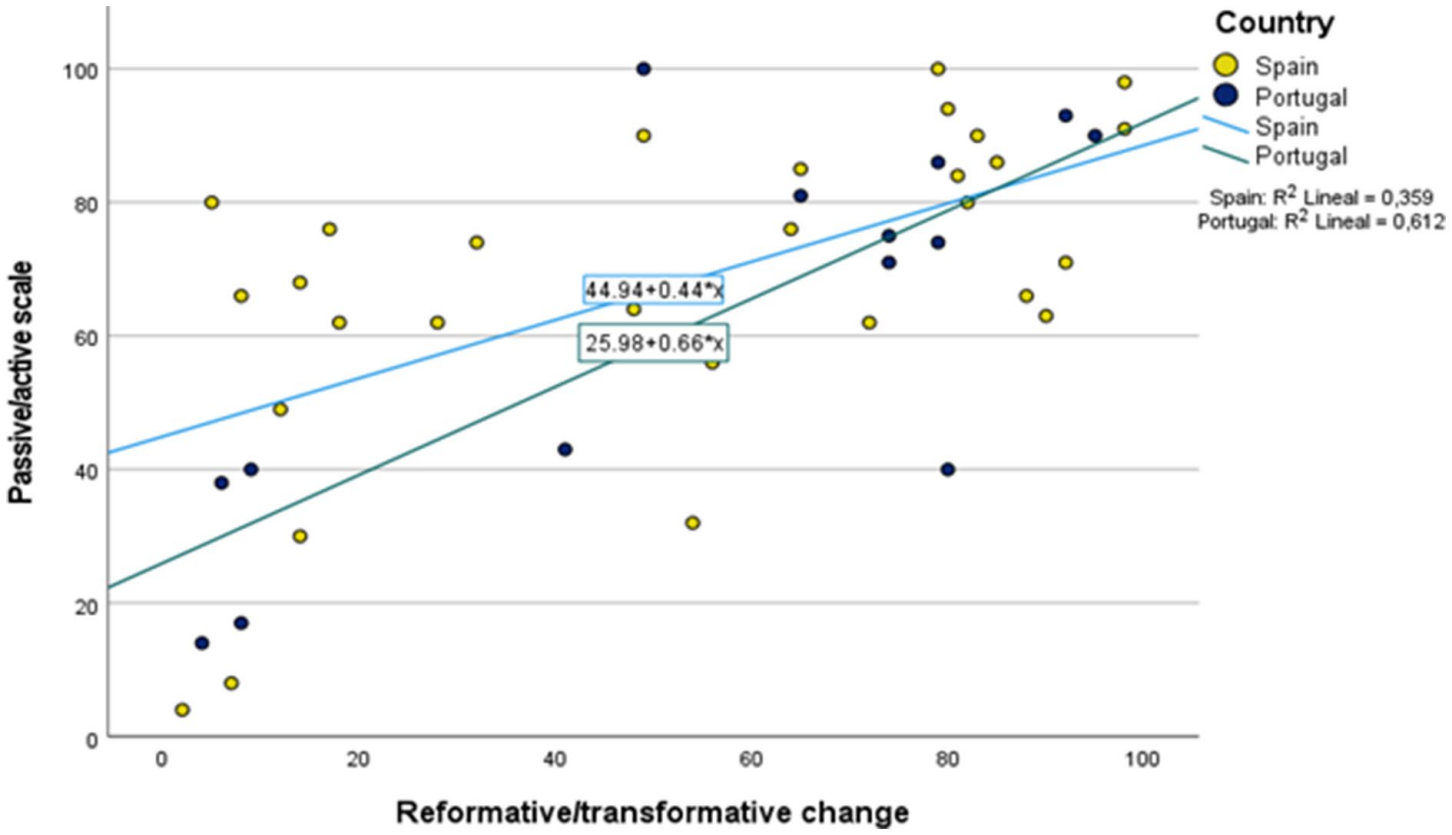
Characteristics of the Spanish and Portuguese cases in relation to their approach to active/passive and transformative/reformative forms of ENCI. Own elaboration.

In relation to their level of approximation to passive/active and reformative/transformative forms, Figure 4 shows the positioning of the cases around these two values along a scale of 0–100 points. The general tendency is towards active (*M* = 65.79, *SD* = 25.66) and transformative (*M* = 52.93, *SD* = 33.19) forms. It was slightly higher the level of active participation of the Spanish citizen in ENCI initiatives (*M* = 67.83, *SD* = 24. 29) compared to Portugal (*M* = 61.57, *SD* = 28.65), and the reverse for the outcomes desired, being slightly more transformative in Portuguese initiatives (*M* = 53.93, *SD* = 34.09) compared to Spanish ones (*M* = 52.45, *SD* = 33.35). Together, it is observed that the more active the initiatives aim to be in both countries, the greater their commitment to transformative outcomes (*r* = 0.656, *p* < 0.001), which is also observed through the fit line in Figure 4 representing the trend of the data based on the regression for each of the two countries.

**Figure 5:**
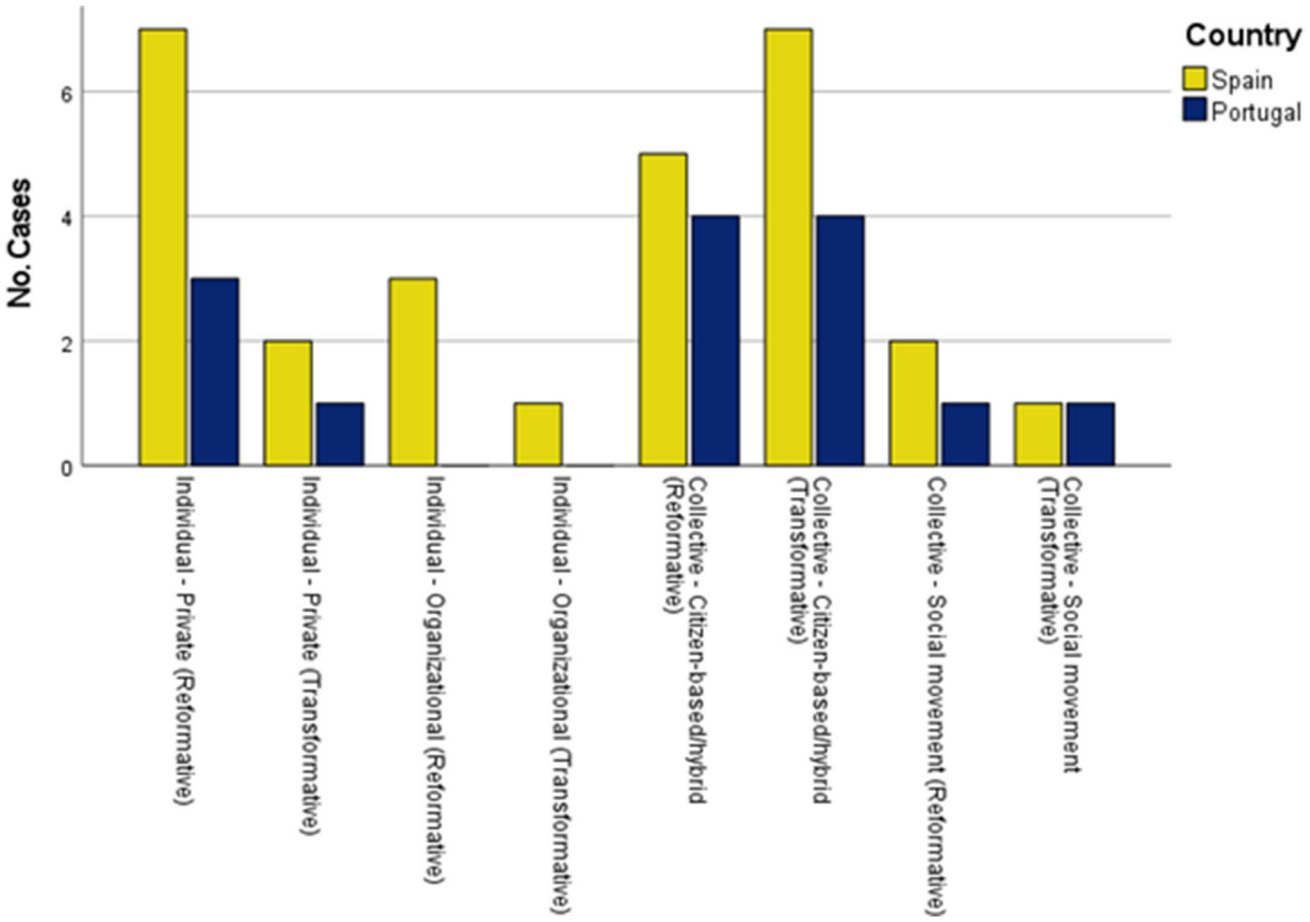
Main type of Spanish and Portuguese ENCI in relation to their private-public/individual-collective distinction. Own elaboration.

In terms of the connection of these initiatives to the wider context, Figure 5 shows the differences in areas of action (private/public and individual-collective).

The main ENCI forms in both countries were the collective ones, with the *citizen-based/hybrid* type standing out, especially ECs and cooperatives (e.g., Pegadas Guimaraes, Coopernico, Valle de las Sensaciones, SomEnergía). These are actions that require a direct involvement of the citizenry, either in its less active and more dependent form on the organisation, as occurs in the reformative vision (e.g., BEHAVE, Luz en Casa Oaxaca, GoiEner), or by promoting radical changes where the citizen is empowered and deeply involved in the changes to be produced in the energy system, as occurs in the transformative perspective (e.g., TRIBE, Banc D’Energia). It should be noted that it is not a question of “all or nothing,” but that there are different degrees, as can be seen in Figure 5.

In the field of individual actions, the reformative private ENCI forms (e.g., Solar Garden, Campanha On–Off) in which the citizen introduces changes in his or her consumption behaviour linked to the purposes of the transition stood out. These are changes that, although they maintain an idea of energy as a “commodity (supply, demand and price are the priority), they also represent necessary options on the road to the energy transition.”

Also, some initiatives were represented in several of the ENCI typologies since their objectives include several actions. For example, in Portugal, the ZERO Association stands as a type of collective movement, which seeks to play an active role in institutional dialogue with the government, with the national and European Parliament and with the different political parties, as well as in regional and local communities, municipal councils and other stakeholders, such as associations and citizens’ movements; but in addition to public action, the members of the association seek to raise awareness and influence in a properly structured and reasoned way, thus gaining credibility in the eyes of society and decision-makers. Another example is On–Off Campaign, which is situated between collective and individual action, since it focuses both on governmental practices that seek to raise awareness by providing individuals with information on consumption, and on individual practices in which each citizen introduces improvements in their consumption patterns at home. In Spain, #NoMásCortesdeLuz platform arose as a social protest movement of diverse actors who call for demonstrations in different cities for promoting energy democracy and social justice, but also refers to individual actors who are called to participate in the signing of the manifesto.

**Figure 6:**
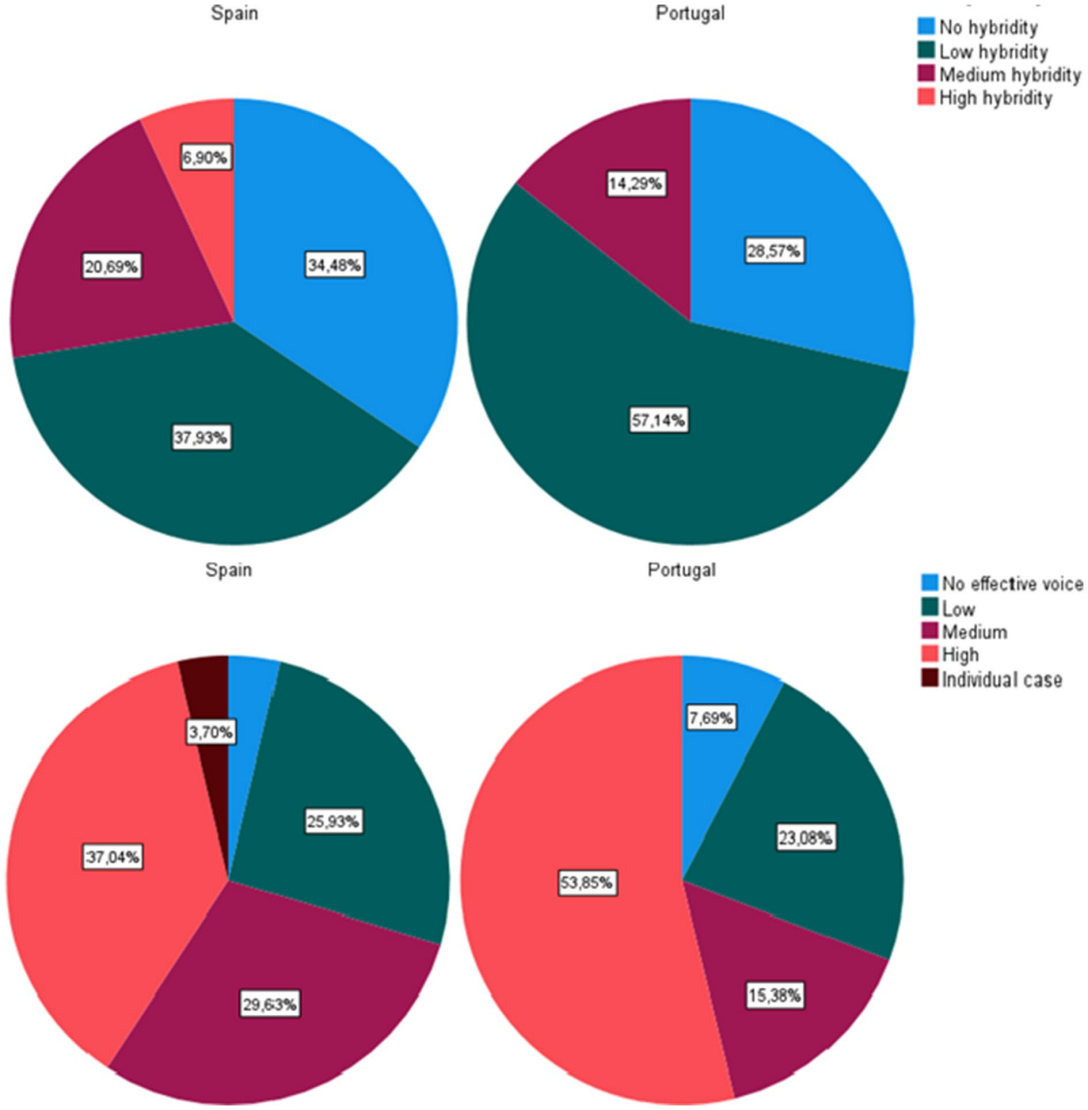
Distinction between Spanish and Portuguese ENCI according to their level of hybridity and effective citizen power/control. Own elaboration.

Finally, regarding the public-private distinction, two other elements define forms of organisation and initiatives closer to what is understood by ENCI, and those are represented graphically in Figure 6.

In the upper part of Figure 6, we refer to the level of involvement of citizens in the institutional sphere of the market and government is referred. Different levels of hybridisation, which still seem to be in incipient stages of development in Southern Europe stood out. Only 6.9% of the initiatives (Spain) reach the highest level, while in most cases in both countries they are in their lowest form. The lower part of Figure 6 shows the proportion of initiatives in which citizens have been shown to have effective power/control within the initiative. In a high proportion - although less than 50% - in both contexts, initiatives have been identified in which the citizen is given a voice, not only as an invited party to the deliberative processes, but as a central axis of the transformative process, sufficiently empowered to exercise control and to make their voice predominate over the rest (e.g., Pegadas Gimaraes; EcoVila).

### 3.2. Results of the stakeholders’ consultation workshop^2^

**Figure 7:**
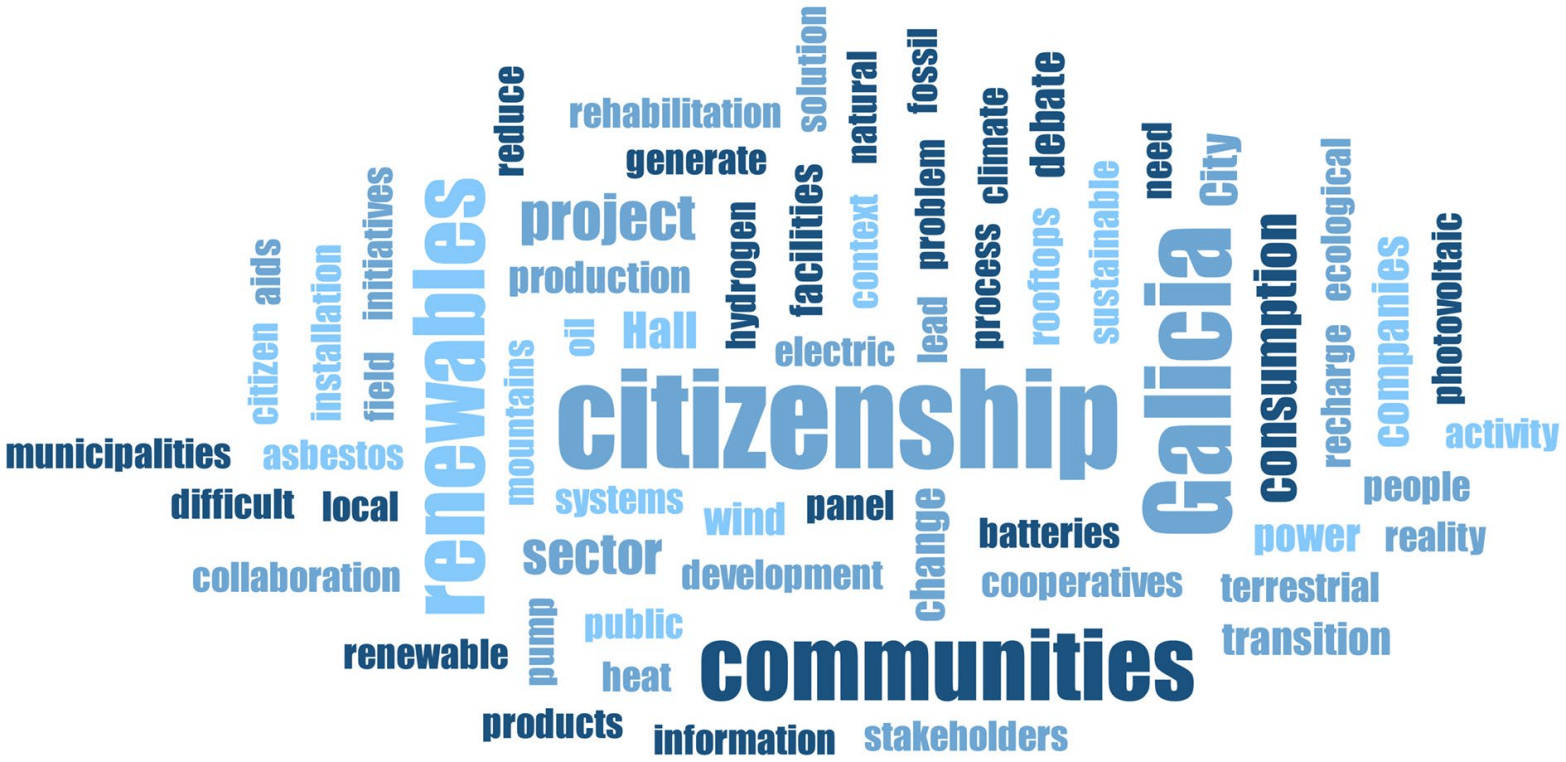
ENCI translations in the Galician context. The colour and size indicate the frequency of occurrence of the concepts.

Considering the results of the initial mapping of initiatives close to ENCI in the Iberian Peninsula and the island territory, it was proposed to go further in validating the typology seeking potential drivers and barriers to their development, from the perspective of various stakeholders. The discussion revealed multiple facets of the concept of ENCI, as shown in Figure 7.

The discourse of stakeholders provided information on the different socio-cultural, political, and economic factors that underlie the development of ENCI types, and how the geographical context may favour or hinder certain forms.

Firstly, in coherence with current European policies that call for a reconceptualization of the role of the citizen, participants stated that a paradigm shift is taking place that can be empowering for individual ENCI types by placing “the citizen at the centre of everything, from a more passive figure, more on the side-lines, to the centre” (A3). The energy citizen is defined as “a person or group of people who are aware of the energy impacts of their daily activity, or even their business” (C1), i.e., in their immediate context; but reference was also made to their key role in bringing about change in the wider energy system, through minimising the economic and environmental impact of energy production and consumption, challenging the current energy system, and as an agent of choice, with options and able to choose for themselves [“the citizen is a true citizen. You can choose, and you have a choice, and you have the ability to choose based on your choices” (B1)]. Indeed, part of the discussion was also devoted to the concept of the individual energy citizen at the level of active energy consumer [“we are now seeing how this citizenship is being empowered and transformed from passive energy consumers to a term that I think is now quite widespread, which is prosumer” (C1)].

Along with the political factor, reference was made to the social factor, in terms of knowledge and access to information available to citizens [“there is misinformation about energy and options” (C2)]. The responsibility for keeping citizens informed and educated lies both with policy makers [“this pedagogy has to come from those who give grants” (B2)] and with citizens and their willingness to transform the current energy system [“everyone will have to become an energy citizen: individual citizens, neighbourhood communities, mountain communities, medium, small and large municipalities, autonomous regions, states and the world, and small, individual, large and very large companies” (A1)].

The economic factor has also been considered a key axis in promoting and limiting the development of ENCI types. Economic support is considered essential for the individual to become an active consumer [“when there are subsidies, people move. There are few people who have enough resources to isolate themselves, for example, if there is no subsidy” (B2)].

Moreover, the development of ENCI types in the Galician territory is not only conditioned by social and structural factors (political and economic), but a key aspect appears in this context: the geographical dimension. The ECs stood out for “the existence and heritage of mountain lands and the creation of joint mountain communities in which they joined forces to minimise the impacts of being isolated” with “public-private collaboration” (A1). The geographical dispersion of the territory, together with political interest, has had a positive impact on the emergence of this collective of ENCI types. Likewise, also derived from the socio-cultural interest it has been possible to recognise latent types, e.g., environmental awareness movements, were also referred to since “we are increasingly aware of what our activity implies and worries us” (A3). In the private sphere, they highlighted “the figure of the prosumer” (B1). It involves individual actions from *some citizens* (e.g., solar panels on the roof; heat pumps; house wrappers; or move to 100% mobility with an electric car…), through the business environment (e.g., membership of companies in industrial estates “through this traditional organizational form that was intended to look after their communal forests”) or under the mediation of local government (e.g., public-private partnerships between companies in the port, with support from the municipality).

The incipient nature of many of the initiatives in a reformative typology was recognized [“what we see is the beginning of initiatives of this type in a phase that is super-embryonic despite the fact that there are already concrete grants or support funds” (C1); “the development of unique projects related to local energy communities” (A2)]. Furthermore, there was pointed out the existence of a base (or heritage) deeply rooted in Galicia of other collective initiatives that “as a social group that is already empowering itself and trying to generate its own energy” (C1) represent “the union of citizens, entities that generate, consume and manage their own energy, in principle, of renewable origin” (A2). Along with this, the role of small and medium-sized enterprises that act as intermediaries [“facilitators and managers for the beneficiary” (A2)] was mentioned, especially in matters of supporting bureaucratic processes that can be an obstacle to the more active involvement of citizens [“the bureaucratic part they demand from us is impossible” (B2)].

In short, energy citizenship is considered a key element in the transition towards a sustainable, fair, and democratic energy system [“precisely the future that awaits us. Energy, which is free, accessible to citizens, but we do not have to buy it, we do not have to manufacture it, we have it at our disposal at any time” (A1)]. Achieving greater energy democracy involves using the SI to find new economic models, new forms of coexistence compatible with a sustainable future that involve “all citizens, communities, small businesses, medium-sized businesses, large businesses” (B1), as well as social models that promote “energy sovereignty, savings and address the problem of energy poverty” (C2).

## 4. Discussion

The purpose of this study was to unpack the empirical manifestations of energy citizenship in the south of Europe. Through desktop research, case analysis and a regional workshop, the ENCI types in Spain and Portugal, examples based on the individual/collective and reformative/transformative typology, as well as facilitating and hindering factors in Galicia have been identified. These points, which respond to the specific objectives of the work, are discussed below.

In response to the first specific objective, the mapping showed a greater presence of initiatives that emerge from citizen interest in the Spanish and Portuguese context (e.g., cooperatives, social protest movements), as well as others that arise from the needs identified by public bodies. In the Spanish and Portuguese contexts, the concern of national and local governments to implement measures that are consistent with the guidelines established by Europe to achieve climate neutrality by 2050 is evident (European Commission, 2019). What these data provide us with is a clearer vision of the role being played jointly by citizens, who are active and interested in adopting a more active role in the energy transition, and political actors, who promote greater citizen participation in democratic and horizontal decision-making processes.

In Spain, the *recognition of the seriousness of climate change* and the *production and/or use of renewable energy* are the main motivations behind energy citizenship initiatives. These data are promising in light of the information provided in the report elaborated by the independent researcher and consultancy Delft (2016), which reveals the enormous potential of renewable projects in the hands of Spanish citizens, with 16.4 million people able to participate in the electricity sector thanks to renewable energies. The initiatives analysed in Spain and Portugal share the motivation to contribute to the energy transition (*n* = 9 cases each), with both countries being in an optimal situation to do so. According to the Energy Transition Index (World Economic Forum, 2021), which compares the energy system performance of different countries, both Spain and Portugal are in the ranking of advanced economies, ranking 17th, and 19th respectively, out of a total of 115 participating countries. This index is not only based on the idea of political and economic development of countries, but also considers the extent to which countries are prepared for the transition to a secure, sustainable, affordable, and reliable energy future. This considers both the design of policies and their social impact, as well as the trust that citizens place in institutions to achieve a just transition.

Thus, the recognition of the existence of socio-environmental problems, along with the trust in the responsible institutions and a willingness to invest in the energy transition are key to achieving greater citizen engagement (Ideara, 2021). From such motivations derive objectives and ambitions such as the *promotion of energy savings, energy justice*, or *carbon footprint reduction efforts*. These results are comparable to those of Campos and Marín-González (2020) who highlighted the relevance given by Portuguese and Spanish initiatives (as well as others from other countries in territorial proximity such as France or Italy) - mainly collective, such as, e.g., local communities - to the link between energy justice and co-ownership in energy generation.

Regarding the second specific objective, a general trend towards active and transformative forms of energy citizenship was found. Specifically, the most active ENCI forms were found in Spain; however, less active and engaged forms, or even non-visible (latent) forms, should not be forgotten as they represent different levels of citizen participation, interest, and empowerment within an initiative and in the wider energy system (Pel et al., 2021). In this sense, the concepts used of low or high energy democracy, active or passive, and reformative or transformative energy citizenship are interpreted along a continuum, as opposed to an “all or nothing” perspective. Within this continuum, several initiatives (e.g., Zero, Smarter Together, La Flor de la Vida) have a high level of citizen participation, while in others the level of citizen participation was lower (e.g., DecoProteste, EcoCasa, Parque Eólico El Hierro). The vision guiding these initiatives is at the basis of these possible differences in levels of participation; from those that aim to implement measures and actions for citizens (e.g., educate and raise awareness on climate change, implement actions by engaging citizens) to those that do so with citizens (e.g., build a more cohesive world, deliver smart and inclusive solutions, contribute to improve self-efficiency). All of them can be meaningful and useful for different energy actors, depending on their individual possibilities (in terms of time, economic, social and knowledge resources, motivation, and interest) and collective possibilities (as a participant and/or member of an initiative) to act in the energy system by making their voice heard in the initiative and beyond it. What is clear is that the higher or lower level of participation have also been related to the reformative or transformative character of energy citizenship initiatives.

A wide look at the set of initiatives allows, therefore, to evidence different forms of citizen involvement, which may offer different results in terms of the capacity to transform the current energy system. Initiatives with a more transformative character and participatory governance structures were more prevalent in Portugal, especially collective initiatives (e.g., energy communities, cooperatives). Energy citizenship initiatives in the south of Europe focus on issues such as energy saving and efficiency, circular economy and the development of research and technological innovation in renewable energies (Campos and Marín-González, 2020). In Spain, there is evidence of an increasing desire to contribute to deep = and radical changes towards environmental sustainability, although reformative initiatives (focusing on incremental changes and less participatory) still predominate. Nevertheless, a problem of some initiatives that have a commitment to deep (transformative) environmental sustainability, shared by the (collective) citizenry, is that they tend to start before regulation is in place, which hinders or diminishes their chances of receiving the necessary economic and social support to develop effectively and/or durably over time (Capellán-Pérez et al., 2018).

Precisely, the analysis of economic and political factors, together with social and geographical factors, was the third goal of the present work. It was found that the appropriate climatic conditions for different renewable energies (e.g., wind), the geographical dispersion of territory and ownership (division in land ownership) and the existence of rural regions without many economic opportunities, but with strong social and cultural roots, have made energy communities to be seen as a very interesting solution by local populations and received the support of public administrations. Indeed, ENCI initiatives were seen as key in the transition from a *model of representative democracy* to a *model based on participatory democratic engagement*, as well as a shift from the idea of passive consumption to *prosumerism* (production and consumption of goods and services), especially when it comes to making improvements or designing living spaces that reduce carbon emissions or encourage self-consumption in individual dwellings. These findings are consistent with the decentralised democratisation model proposed by Thombs (2019), which showed that the democratisation of the social (political, economic, and civil spheres) can lead to democratic outcomes (participatory, associative, and deliberative processes), justice (in the distribution of cost-benefits, social representation, and access to decision-making) and ecological outcomes (reduction of energy use and GHG emissions). However, it has become evident how the development of ENCI initiatives can be constrained by issues such as rural communities’ distrust of alternative land use initiatives, or concerns related to energy markets, instabilities, political systems, among others, which hinder the acceleration of energy transformations and ultimately affect citizens’ decisions on their commitment to the transition towards democratic and socially responsible forms of energy.

The discourse of representatives of the Government, Business and intermediaries for the Civil Society has revealed the confrontation in relation to the responsibility for the slow development of these initiatives in the Galician territory. The business and social sector has criticised the lack of local regulation, and the insufficiency of urban planning and citizen information, while the local administration points out that what is lacking is a change of mentality and support from local agents, given the limitations that local governments have in terms of human, technical and economic resources. It is of great concern to note the reluctance of the population to trust public institutions (Ideara, 2021), especially if we consider that, as we pointed out in response to objective 1, a large part of the initiatives is promoted by public authorities. Additional data is provided by Eurobarometer (2022) No. 527 which refers to the lack of trust in regional, municipal, and local authorities (54% Spain; 49% Portugal) and in the government (57% Spain; 49% Portugal).

Hence the need to be cautious about urging citizens to take responsibility and raise expectations about their contribution to the energy transition, when they may not be able to do so, which can have a disempowering effect (Lennon et al., 2020). In the Galician case, there seems to be a strong interest of citizens to act in an energy sustainable way in their homes (e.g., solar panels, purchase of electric vehicles, or replacement of the boiler by heat pumps) and a huge potential for citizens to be autonomous and independent in their energy management and consumption. But when these individual forms are not backed up by proper regulation, and by capacity building and financial support from state/public institutions, success is limited.

## 5. Conclusion

ENCI refers to a broad, complex construction, loaded with interpretations dependent on political, social, cultural, and economic factors, and it is not possible to refer to a single type of initiatives or to an *optimal* level of energy citizenship, but to varied forms influenced by elements of awareness, motivation and concern for the energy system and its consequences on climate change.

This study has sought to shed light on this question by showing that, under different political ideals of ENCIs, particular forms of active, engaged, and empowered citizenship are assumed. However, it is not possible to refer to a single type of ENCI initiatives or to an *optimal* level of energy citizenship, but rather to varied forms influenced by different sets of individual, social and political conditions. The mapping of initiatives in Spain and Portugal has revealed diverse manifestations of energy citizenship that, more overtly or latently, show a commitment to move towards a governance model where power and control are in the hands of citizens, where change occurs in the wider energy system, and where there is a strong commitment to sustainability, democracy, and energy justice. Achieving profound transformations in values, belief systems and relationships with the environment and between individuals will require, on the part of these initiatives, a continuous effort of creativity to offer innovative responses adapted to the relational and organisational conditions of each geographical context, together with the search for more active citizen participation in building consensus around the objectives and priorities of Spanish and Portuguese societies in the framework of the energy transition.

What can be observed in Southern Europe is an incipient development of citizen-based initiatives actively engaged in the development of profound changes in the energy system. In this respect, a predominance of collective forms and, to a lesser extent, incipient forms of individual and private reform actions were found. Regarding the classification of active-passive ENCI in this study, some caution is recommended in its interpretation so as not to run the risk of limiting our knowledge by “framing citizens’ actions in a single category” (Pel et al., 2021, p. 20). On the contrary, under the different political ideals of the ENCIs, particular forms of active, engaged, and empowered citizenship are assumed. Similarly, different levels of deepening or questioning of existing structures have been observed between the initiatives analysed in Spain and Portugal. The existence of socio-environmental problems (Ideara, 2021; Linares et al., 2022) awakens in people feelings of indignation and injustice that can materialise in collective actions against those responsible for the environmental problem, for example, public authorities (Eurobarometer, 2022). Additionally, the study of the Galician region revealed the presence of facilitating and limiting factors at the political and economic, geographical, and socio-cultural levels. This has been key to a deeper understanding of the predominant presence of collective and reformative types in Southern Europe.

Some limitations deriving from the methodology employed should be highlighted. As one of the criteria for the mapping is the availability of sufficient information on the initiative, it is possible that many valuable initiatives have not been considered, and especially individual forms of energy citizenship were not sufficiently included. Future research should also more in-depth research on the mapped initiatives could delve deeper into the motives behind citizens creating or joining ENCI initiatives, the level to which they feel they have autonomy, capacity, and control over the energy decisions they make, as well as the resources available to them and conferred by the initiative itself to act in the wider system.

## Data availability statement

The original contributions presented in the study are included in the article/Supplementary material, further inquiries can be directed to the corresponding author.

## Author contributions

All authors listed have made a substantial, direct, and intellectual contribution to the work, and approved it for publication.

## Funding

This project has received funding from the European Union’s Horizon 2020 research and innovation programme under grant agreement no. 101022492.

## Conflict of interest

The authors declare that the research was conducted in the absence of any commercial or financial relationships that could be construed as a potential conflict of interest.

## Publisher’s note

All claims expressed in this article are solely those of the authors and do not necessarily represent those of their affiliated organizations, or those of the publisher, the editors and the reviewers. Any product that may be evaluated in this article, or claim that may be made by its manufacturer, is not guaranteed or endorsed by the publisher.

## Footnotes

^1^A complete table is presented as supplementary material. It presents a general overview of the mapping carried out in the two contexts studied, incorporating descriptive information on the main characteristics of each initiative.

^2^The coding used for the quotations presented here refers to officials from local (A1), regional (A2) and a regional delegation of a national center (A3), representatives of an energy supply company (B1) and a small construction company (B2) and representatives of an R&D center (C1), and of a non-profit association (C2).
