# Supplementary material for: Mapping energy citizenship in the south of Europe
Source: Front Psychol. 2023 Aug 9;14:1112457. doi: 10.3389/fpsyg.2023.1112457 (PMC10445128; doi:10.3389/fpsyg.2023.1112457)
Supplement: Supplementary file 1 [file Table_1.docx]

Supplementary Material

**Table (supplementary material)**

*Main characteristics of the Spanish and Portuguese initiatives included in the mapping.*

| **Country** | **Focus** | **Agency** | **Target Area** | **Title** | **Description** | **Motivations to start** | **Main objectives** | **Start / End date** |
| --- | --- | --- | --- | --- | --- | --- | --- | --- |
| PT | Holistic | Collective | Several types | ZERO | Association born from the common interest of a hundred people to achieve sustainable development in Portugal. It seeks the proactive participation of citizens, to build a more cohesive world, socially and economically, in full respect of the natural limits of the planet. | - Contribute to the energy transition - Discontent because energy transition is not going fast enough | - Energy justice - Energy democracy - Creating/promoting an alternative societal and economic model. | 2011-2015 |
| SP | Holistic | Collective | Virtual case | BEHAVE | Project aimed to enhance the performance of energy-related behaviour change programmes by adopting a rigorously scientific approach to evaluate recent examples, and by developing an effective model for design, implementation, and evaluation of this type of programmes for use by policy makers, programme designers/managers, and consumer organisations. | - Contribute to energy transition. - Increasing public involvement | - Promoting energy saving - Action aimed at altering proposed projects of others - Creating/promoting an alternative societal and economic model | 2006-2010 / Earlier than 2015 |
| SP | direct energy production/ consumption | Collective | Virtual case | TRIBE: | Project that develops a new energy efficiency videogame, running with real data from public buildings. The game includes the different relevant aspects for improving the energy efficiency in buildings. | - Increasing/achieving self-sufficiency - Contribute to energy transition - Availability of incentives | - Promoting energy saving - Reducing the carbon footprint - Creating strategic intelligence via networking activities and products | 2011-2015 / 2018 |
| SP | Holistic | Collective | Peri-urban | Banc d’Energia | A legally constituted association that promotes energy saving and efficiency for the benefit of those in a situation of energy vulnerability. This is done through pedagogy and transformative solidarity. | - Recognition of the seriousness of climate change - Realization of a particular energy related injustice | - Energy justice. - Energy democracy - Alleviating energy poverty | 2016-2020 / 2018 |
| PT | Holistic | Collective | Urban | PEGADAS | A transversal program, dedicated to environmental education, constituting a fundamental factor for the increase of community practices based on ecologically sustainable principles. | - Contribute to the energy transition. - Discontent because energy transition is not going fast enough | - Promoting/enabling climate action | 2016-2020 |
| PT | direct energy production/ consumption | Collective | Several types | Campanha On-Off | Agency that, in collaboration with RTP (national TV) run an energy efficiency awareness raising campaign targeting families. A series of short movies was produced, explaining the concepts, and giving tips to the public. | - Contribute to the energy transition - Discontent because energy transition is not going fast enough. | - Promoting/enabling climate action - Promoting energy saving - Reducing the carbon footprint | 2016-2020 |
| PT | mobility | Collective | Urban | Park4SUMP | Project that defines parking management models in different European cities to ensure a holistic approach to the different areas of mobility that encourages more sustainable lifestyles, less dependent on traditional motor vehicles. | - Contribute to energy transition. - Need to respond to national demand | - Ending dependence on fossil fuels - Reducing carbon footprint - Creating/promoting an alternative societal model | 2016-2020 |
| PT | direct energy production/ consumption | Collective | Urban | SOLIS | An initiative that proposes to design a solar map (cartography) to provide citizens, local authorities, investors, and companies with a series of attractive data and graphics, at city, neighbourhood and building level. | - Contribute to the energy transition - Increasing public involvement | - Ending dependence on fossil fuels - Creating and promoting an alternative societal model | 2016-2020 |
| SP | Holistic | Collective | Several types | No Mas Cortes de Luz | The #NoMásCortesDeLuz campaign was created to denounce power cuts to families without resources and the abusive tariffs of the electricity sector. | - Realization of a particular energy related injustice - Frustration due to lack of action by decision makers - Need to respond to national demand | - Alleviating energy poverty - Lobby for an institutional act - Creating/promoting an alternative societal model | 2011-2015 |
| SP | Holistic | Collective | Peri-urban | EOLPOP | Pioneering initiative for the installation of a wind turbine with shared ownership among citizens who voluntarily contribute capital. It is located in the municipality of Pujalt (Anoia, Catalonia) due to the good wind conditions, easy access and accessibility to the medium voltage grid. | - Contribute to the energy transition - Produce/use renewable energy - Increasing public involvement | - Promoting/enabling climate action - Energy justice - Energy democracy | 2006-2010 |
| SP | direct energy production/ consumption | Collective | Several types | Auditorías Energéticas | Project focused on improving energy efficiency and savings in households (thermal and electrical, e.g. possible heat losses, characteristics of domestic electricity) throughout Galicia, Ibiza, La Rioja, Mallorca and Madrid. | - Recognition of the seriousness of climate change - Contribute to the energy transition - Produce/renewable energy | - Promoting/enabling climate action - Energy justice - Reducing the carbon footprint | 2011-2015 / 2016 |
| PT | direct energy production/ consumption | Collective | Several types | EnergizAir | Project reporting positive indicators on the share of energy needs covered by renewable energy sources in the weather forecast. | - Contribute to the energy transition - Produce/use renewable energy - Increasing public involvement | - Supporting, promoting, enabling prosumerism - Promoting energy saving | 2006-2010 / earlier than 2015 |
| PT | Holistic | Collective | Virtual | Deco Proteste | Renewable energy community designed to keep the consumer informed to make informed decisions on the purchase of renewable energy solutions (RES) | - Produce/use renewable energy - Increasing/achieving self-sufficiency | - Reducing the carbon footprint - Increasing/achieving self-sufficiency - Promote/defend consumer interests, problems and rights. | 2011-2015 |
| SP | direct energy production/ consumption | Collective | Peri-urban | Huerta Solar | Project that offers people the chance to become co-owner of a photovoltaic installation on a roof by financial holdings. The electricity generated is supplied to buildings nearby through the grid to avoid energy losses. | - Inspiration by a similar case elsewhere - Produce/use renewable energy | - Promoting/enabling climate action - Energy justice - Increasing/achieving self-sufficiency | 2001-2005 |
| SP | direct energy production/ consumption | Collective | Several types | Energía Comunitaria | An association without financial motives that seeks to create a local and global change towards a more sustainable society. Based in Madrid, partners in 70 counties (global perspective). Promotes community renewable energy generation projects (PV and wind), putting it in the hands of the citizen. | - Recognition of the seriousness of climate change - Realization of a particular energy related injustice - Discontent because energy transition is not going fast enough | - Promoting/enabling climate action - Energy justice - Protest for the energy transition | earlier than 1992 |
| SP | Holistic | Individual | Rural | Valle De Las Sensaciones | A prototype of an Ecovillage Laboratory, whose focus is an experiential integration of humans into nature. Founded by a couple being fed up with mainstream unsustainable practices. | - Frustration due to lack of action by decision makers - Need to respond to local/national/etc. Demand | - Supporting, promoting, enabling prosumerism - Increasing/achieving self-sufficiency - Creating/promoting an alternative societal model | 2001-2005 |
| PT | Holistic | Collective | Rural | Eco-comunidades | Green communities designed to connect families and concerned citizens to create low-carbon lifestyles (think globally, act locally), followed by a local strategy, also applicable at the national level. | - Recognition of own responsibility - Recognition of the seriousness of climate change | - Ending dependence on fossil fuels - Promoting energy saving - Reducing the carbon footprint | 2011-2015 |
| PT | direct energy production/ consumption | Collective | Several types | Coopernico | Renewables cooperative, which gives support to solidarity, educational and environmental protection projects. | - Produce and/or use renewable energy | - Supporting, promoting, enabling prosumerism - Energy democracy - Increasing/achieving self-sufficiency | 2011-2015 |
| SP | direct energy production/ consumption | Individual | Rural | Casita Verde | Experiential learning centre, designed to encourage innovative and sustainable lifestyle techniques, within the reach of each of us. | - Recognition of own responsibility | - Supporting, promoting, enabling prosumerism - Increasing/achieving self-sufficiency | 1992-1995 |
| SP | direct energy production/ consumption | Collective | Rural | Luz en Casa Oaxaca | Rural Electrification Programme adapted by the ACCIONA Foundation to provide a solution to communities with <100 inhabitants in Oaxaca (Mexico), where the public electricity company had no electrification plans. | - Realization of a particular energy related injustice | - Alleviating energy poverty | 2011-2015 |
| SP | Holistic | Collective | Several types | Energía con conciencia | Educational project of Fundación Repsol to make young people aware of the importance of responsible use of energy and the value of existing resources. | - Recognition of own responsibility - Recognition of the seriousness of climate change | - Promoting energy saving - Increasing/achieving self-sufficiency | 2011-2015 |
| SP | Holistic | Collective | Urban | Madrid 100% Sostenible | Citizen-led movement in Madrid supported by Alianza por el Clima (400 organizations), committed to guaranteeing municipal contracts for 100% renewable energy, implementing energy efficiency programmes in schools, and establishing fiscal measures to promote energy efficiency. | - Contribute to the energy transition - Discontent because energy transition is not going fast enough - Increasing public involvement | - Promoting energy saving - Energy justice | 2016-2020 |
| SP | direct energy production/ consumption | Collective | Several types | GoiEner | Non-profit cooperative that believes that electricity is now a need as basic as food, and wants consumers to reclaim their energy sovereignty and make them aware of its importance. | - Discontent because energy transition is not going fast enough - Produce/use renewable energy | - Supporting, promoting, enabling prosumerism - Ending dependence on fossil fuels - Energy democracy | 2011-2015 |
| SP | direct energy production/ consumption | Collective | Several types | SomEnergia | Non-profit green energy consumer cooperative, whose main activities are the marketing and production of energy from renewable sources. They are committed to driving a change in the current energy model to achieve a 100% renewable model. | - Discontent because energy transition is not going fast enough - Produce/use renewable energy | - Supporting, promoting, enabling prosumerism - Ending dependence on fossil fuels - Energy democracy | 2011-2015 |
| PT | Holistic | Collective | Several types | APREN | Non-profit association founded in October 1988, with the mission of coordination, representation and defence of the common interests of its Members. | - Contribute to the energy transition - Produce/use renewable energy - Need to respond to local/national/etc. Demand | - Supporting, promoting, enabling prosumerism - Ending dependence on fossil fuels - Reducing the carbon footprint | earlier than 1992 |
| PT | direct energy production/ consumption | Collective | Several types | EuroTopten Act | Project aimed at helping the Portuguese consumer to find the most efficient domestic machines on the market. The project is part of Horizon 2020. In PT it is led by Quercus and ADENE. | - Recognition of own responsibility - Discontent because energy transition is not going fast enough | - Promoting energy saving | 2006-2010 |
| SP | direct energy production/ consumption | Collective | Several types | USmartConsumers | Project aimed at promoting the correct use of smart meters by consumers and the development of new services related to smart meter by operators energy | - Recognition of own responsibility - Availability of incentive | - Promoting energy saving - Creating strategic intelligence via networking activities and products | 2011-2015 / 2017 |
| SP | direct energy production/ consumption | Collective | Several types | Piscinas Solares | Company that sells purified water using electricity produced by solar energy. | - Contribute to the energy transition - Produce/use renewable energy | - Supporting, promoting, enabling prosumerism - Increasing/achieving self-sufficiency | not known |
| SP | Holistic | Collective | Rural | Parque Eólico de El Hierro | First island to secure a constant supply of electricity by combining wind and waterpower and with no connection to any outside electricity network | - Contribute to the energy transition - Produce/use renewable energy | - Ending dependence on fossil fuels - Reducing the carbon footprint | 2011-2015 |
| SP | Holistic | Collective | Several types | Euronet 50/50 | Project aimed at mobilising energy savings by applying the 50/50 methodology in 500 schools and almost 50 public buildings in 13 EU countries (114 of SP). Schoolchildren learn how to be more energy efficient and the importance of doing so, and take this information back to families. | - Recognition of own responsibility - Contribute to the energy transition - Produce/use renewable energy | - Promoting energy saving - Reducing the carbon footprint | 2011-2015 |
| SP | direct energy production/ consumption | Collective | Several types | Liberar Al Sol | Proposal of an easy "10 step guide" to empower citizens to produce and consume their own energy in a collective form, as well as to reduce their consumption through energy savings and efficiency. | - Recognition of the seriousness of climate change - Realization of a particular energy related injustice | - Promoting/enabling climate action - Energy justice | earlier than 1992 |
| SP | direct energy production/ consumption | Collective | Rural | Noctisolar Ecolight | Project in collaboration with a company in the sector T-SOL and the Terra Foundation, to provide an answer to the problem of domestic lighting in areas without electrification, offering low-cost, high-efficiency solar technology through a portable solar lamp. | - Recognition of own responsibility - Realization of a particular energy related injustice - Need to respond to local demand | - Alleviating energy poverty | 1996-2000 / earlier than 2015 |
| SP | Holistic | Collective | Peri-urban | La Flor de la Vida | Innovative project of sustainable urban growth, based on a natural geometric pattern. The concept of this sustainable Ecovila allows the development of an economy that respects human beings, where the project itself marks a new paradigm of economic efficiency. | - Recognition of own responsibility - Community building - Need to respond to local demand | - Ending dependence on fossil fuels - Promoting energy saving - Increasing/achieving self-sufficiency | 2011-2015 |
| SP | Holistic | Collective | Urban | Granada en transición (GET) | Project initiated by a group of people from Granada (SP) to create a portal for support and dissemination of initiatives that face current challenges such as climate change, economic and social crisis, inequalities and dependence on fossil fuels and their derivatives. | - Recognition of own responsibility - Inspiration by a similar case elsewhere - Community building | - Promoting energy saving - Reducing the carbon footprint - Increasing/achieving self-sufficiency | 2011-2015 |
| PT | Holistic | Collective | Several types | Energia e Alterações Climáticas (Quercus) | The Energy and Climate Change area of the Quercus Foundation works on the environmental education of citizens and the monitoring of public policies. | - Recognition of the seriousness of climate change - Contribute to the energy transition - Increasing public involvement | - Promoting and enabling climate action - Promoting energy saving - Encouraging, enabling debate on energy and/or climate issues | earlier than 1992 |
| SP | direct energy production/ consumption | Collective | Several types | Hogares Verdes | Educational program born in Segovia from Centro Nacional de Educación Ambiental to help families by promoting autonomy in domestic consumption of water and energy and helping them make ethical purchases | - Recognition of the seriousness of climate change | - Promoting energy saving - Reducing the carbon footprint | 2006-2010 |
| SP | Holistic | Collective | Urban | Smarter Together | Project that seeks the right balance between smart technologies and the organisational and governance dimensions to deliver smart and inclusive solutions and improve citizens' quality of life. | - Contribute to the energy transition | - Promoting energy saving - Energy democracy - Creating/promoting an alternative societal model | 2016-2020 / 2021 |
| SP | direct energy production/ consumption | Collective | Several types | Sol sin límites, energía sin límites | Environmental education program for schools in 16 provinces about the use of solar energy as a clean energy source. Created by Fundación Oxígeno, with financial support of Obra Social Caja Madrid. | - Recognition of the seriousness of climate change | - Promoting energy saving - Reducing the carbon footprint | 2006-2010 |
| PT | Holistic | Collective | Rural | Tamera | The Healing Biotope I Tamera is a research project with the goal to create the model for a future society that is free from hatred, lies, violence and fear. | - Community building - Contribute to energy transition | - Creating/promoting an alternative societal model | earlier than 1992 |
| PT | direct energy production/ consumption | Collective | Several types | EcoCasa | The project works on Environmental Awareness/Education to save natural resources in our homes | - Recognition of the seriousness of climate change | - Promoting energy saving - Reducing the carbon footprint | 2001-2005 |
| SP | Holistic | Collective | Urban | La Borda | Housing cooperative for the transfer of use, under a non-speculative model. It aims to prioritise the environmental aspect, economically achievable through passive design or low energy consumption housing, with local, decentralised, and self-managed generation of renewable energies. | - Inspiration by a similar case elsewhere - Community building | - Creating/promoting an alternative societal and economic model | 2011-2015 |
| SP | Holistic | Collective | Rural | Proyecto o Couso | An integrated and open community where everyone operates under the principle of "Leave what you can; take what you need." The self-sufficient ecovillage has many permanent residents and hosts pilgrims making the Camino de Santiago. | - Unknown/cannot be defined at this stage | - Creating/promoting an alternative societal and economic model | 2011-2015 |
| SP | direct energy production/ consumption | Collective | Rural | 8th Life EcoVillage | Project launched by the NPO/NGO Gaia Tasiri Association to repopulate a rural hamlet and establish a community for more effective work in facilitating global and local transition and action research, organised around ecology and sustainability. They are a self-described Transition Town (post-oil and off-grid communities). | - recognition of own responsibility | - promoting energy saving - energy justice | 2006-2010 |
